# Jumping Characteristics of Broiler Breeder Hens at Different Perching Platform Heights

**DOI:** 10.3390/ani15050725

**Published:** 2025-03-03

**Authors:** Xiaoliu Xue, Baoming Li, Qin Tong, Yang Wang, Peng Yin

**Affiliations:** 1 Department of Agricultural Structure and Bioenvironmental Engineering, College of Water Resources and Civil Engineering, China Agricultural University, Beijing 100083, China; xuexiaoliu@cau.edu.cn (X.X.); tongqin@cau.edu.cn (Q.T.); wangyang512@cau.edu.cn (Y.W.); yinpeng@cau.edu.cn (P.Y.); 2 Key Laboratory of Agricultural Engineering in Structure and Environment, Ministry of Agriculture and Rural Affairs, Beijing 100083, China; 3 Beijing Engineering Research Center on Animal Healthy Environment, Beijing 100083, China

**Keywords:** platform heights, jump timing, jump behavior, jump displacement, jump angle

## Abstract

Excessive mating is a significant welfare concern for floor-reared broiler breeder hens. Perches have shown potential as a solution to mitigate this issue; however, limited research is available on the design parameters of perches. Understanding the jumping characteristics of broiler breeder hens is crucial for designing safe and functional perches. In this study, perching platforms were selected to ensure the safety of hens during jumps. Videos of hens performing voluntary jumps from varying heights (35, 40, 45, and 50 cm) were analyzed using Tracker (version 6.2.0) software. The results showed that the take-off latency (*TL*) and jump duration (*JD*) increased with height increases, with downward jumps exhibiting a 66.1% and 10.7% higher *TL* and *JD* than those of upward jumps (based on mean values), respectively. However, the latency to achieve balance (*LAB*) decreased by 76.5% during downward jumps (based on mean values). During upward jumps, both the wing-beating frequencies during the jump (WBJD) and after landing (WBL) increased with height increases. The interaction between the height and direction of jumping significantly affected the horizontal displacement (body) and angles (body and head) during the JL phase (*p* < 0.05). The body horizontal displacement during downward jumps was up to 70.3% higher than that during upward jumps (based on mean values), while the downward body angles were more minor than those during upward jumps. The lowest body and head angles of downward jumps were 42.2° ± 5.4° and 33.4° ± 5.6°, respectively. The results related to TL and LAB are expected to provide guidance for designing appropriate heights in perching facilities. Meanwhile, the findings on horizontal displacement and jump angles offer valuable insights for the safe distance and angle design between perching platforms.

## Simple Summary

Broiler breeder hens raised in natural mating systems often experience excessive mating, which can negatively impact their welfare. Providing structures such as perching platforms may help alleviate this issue. However, limited research has been conducted on designing safe and functional perching platforms. Understanding how hens jump between perching platforms is essential for optimizing their design. In this study, we observed and analyzed voluntary jumps from different heights to assess movement patterns. The results showed that as the jump height increased, the hens took longer to initiate and complete their jumps, particularly for downward movements. However, they regained balance more quickly after landing from higher jumps. The wing movements varied with both the height and jump direction, with downward jumps exhibiting greater horizontal displacement and smaller body and head angles compared to upward jumps. These findings provide important insights into how broiler breeder hens move and balance themselves when jumping. The results can help improve perch design, ensuring better safety and comfort for hens in commercial poultry systems, ultimately enhancing their welfare.

## 1. Introduction

With increasing public attention to animal welfare, improving the welfare standards of poultry farming has become a significant research focus. In natural mating systems, excessive and aggressive mating in broiler breeder flocks threatens the health and welfare of hens [1,2,3,4]. Elevated slatted areas and nesting boxes can serve as refuges for injured broiler breeder hens [5]. At the same time, the provision of perches and multi-tier systems offers hens spaces to escape excessive mating, thereby improving mating behaviors [6]. Furthermore, previous studies have highlighted the importance of perches as a key welfare facility in poultry farming systems. Perches not only meet the roosting needs of poultry [7] but also project them from predators [8] and help reduce feather and footpad injuries [9,10], contributing significantly to poultry welfare. For this reason, perches are widely adopted in layer farming systems. However, perches are often absent in broiler breeder farming systems [11,12,13]. This may be due to differences in size and body weight between broiler breeders, parent stocks of high-growth-rate broilers [14], and layers, potentially affecting their ability to utilize perches effectively.

Several studies have demonstrated that broiler breeders possess the ability to utilize perches. Gebhardt-Henrich et al. [12] found that despite being constrained by body weight, Ross 308 and Sasso broiler breeders can use perches, and their perch use follows a similar temporal pattern to that of laying hens. Broiler breeders show a preference for using Siesta perches at a height of 15 cm [15], and Ross 308, Ross 708, and Ross Ranger broiler breeders are even capable of perching on wooden perches at a height of 47.5 cm [16]. However, despite their ability to use perches, broiler breeders’ relatively high body weight imposes greater demands on the design of perching facilities, particularly in ensuring the safety of jumps.

Animal welfare depends on its ability to adapt and thrive harmoniously with its environment, ensuring physical and psychological well-being [17]. The design of perches is closely related to the landing accuracy, safety, and welfare of poultry during inter-tier movements [18,19,20]. As the crucial expression of poultry movement between multi-tier perches, jumping characteristics directly reflect whether the design of perching facilities meets their behavioral needs. Therefore, exploring the jumping characteristics of broiler breeder hens between differently designed perches is of great significance for optimizing perching facility design, reducing hen stress, and improving welfare. Previous studies on perch design for laying hens have shown that factors such as the jump direction, distance, and angles between perches can affect the landing accuracy [21]. Additionally, the jumping characteristics of laying hens concerning the variations in perch height [22], horizontal distance [23,24,25], and angle [22] have been explored. However, relevant research data on broiler breeder hens are lacking. The comprehensive effects of different perch heights on the jump timing, wing-beating frequencies, horizontal displacement, and jump angles of broiler breeder hens remain unclear, making it challenging to optimize perching facility design parameters in practical production precisely. Moreover, previous studies often investigated perching facility design parameters by exploring the jumping characteristics of laying hens between perches with fixed positional relationships [26]. However, this may pose challenges for broiler breeder hens. These challenges may lead to a large gap between how different design factors affect jumping characteristics in broiler breeders and existing research on jumping characteristics in laying hens.

The present study aimed to fill this gap by systematically analyzing the jumping characteristics of broiler breeders at different perching platform heights (35, 40, 45, and 50 cm). This study focused on jumping characteristics, including jump timing, wing-beating frequencies, horizontal displacement, and jump angles, aiming to reveal the effects of the perching platform height and jump direction on these characteristics. Based on these metrics, our research hypotheses were as follows: the jumping time (take-off latency (TL) and latency to achieve balance (LAB)) will increase with increased perching platform height because higher perching platforms may require more time for the hen to make adjustments before and after the jump, and the associated results will guide the design of appropriate heights between different perching platforms. The wing-beating frequency will be higher on higher perching platforms because hens will need more wing assistance to navigate longer distances. Horizontal displacement and jump angles can vary with perching platform height, and the associated results will guide the design of safe distances and angles between perching platforms.

## 2. Materials and Methods

### 2.1. Animals and Housing Management

Roosters and hens of Arbor Acres parent broiler breeders were transferred from the brooder farm and placed in a two-high and one-low natural mating house at a separate poultry house at week 8; they could move freely between the litter and slatted areas. During week 18, hens and roosters were allocated to the experimental house at a ratio of 9:1~10:1. Black plastic netting was used to confine the hens and roosters to the slatted and litter areas, respectively. After one week, the partitions were removed, allowing the hens and roosters to move freely between the slatted and litter areas, completing the mixing process. At the start of the experiment, there were 6000 experimental poultry-housed hens and 566 roosters. The detailed nutritional composition of the feed provided to the AA+ broiler breeders is outlined in Supplementary Table S1.

The experimental house was 90 m long and 15 m wide, containing a litter area of 3.68 m width and a slatted area of 5.66 m width and 30 cm height on both sides. Therefore, the broiler breeders in the experimental enclosures had prior experience using a 30 cm high space during the early stages of the study. There were egg-laying boxes, water lines, and feed lines in slatted areas for hens. There were rice hulls with a depth of about 5 cm and feed lines in the litter area for roosters. The light program was 14 h of light (05:30 to 19:30) and 10 h of darkness (19:30 to 05:30). During the trial, hens had immediate access to feed upon light onset, whereas roosters were manually fed after the lights were turned on. Water was freely available to all breeders throughout the light period.

In this study, we did not specifically control for body weight differences among the observed broiler breeder hens. However, the experiment was conducted in a commercial poultry production environment, where producers regularly monitor and identify hens with abnormal weight (either too high or too low) and adjust their feeding accordingly to maintain weight uniformity within the flock. Therefore, although body weight was not a direct variable in this study, the weight distribution within each enclosure remained stable and consistent throughout the experiment.

### 2.2. Setup of the Testing System and Video Data Collection

**Figure 1:**
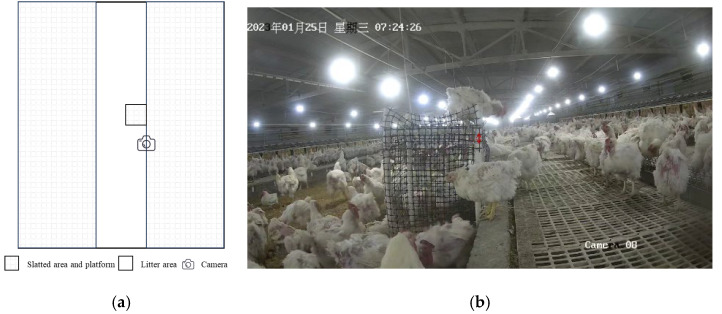
Testing system for analyzing the jumping characteristics of hens: (**a**) Schematic diagram. (**b**) On-site setup. The two ends of the red double arrow point to two parallel marker lines. Screenshot taken on 25th January 2023 at 7:24:26, as indicated by the date and time in Chinese characters.

In three experimental enclosures (each measuring 18 m in length and 15 m in width), one testing system, as shown in Figure 1, was installed in each enclosure. Each testing system, consisting of a perching platform and a camera, was fixed at the boundary between the litter and slatted areas, forming a controlled observation area within the larger enclosure. Each perching platform measured 50 cm × 50 cm (length × width), with its top parallel to the ground. The base was enclosed with black plastic netting, and the vertical rods were marked with parallel lines at 5 cm intervals using a black marker for distance reference.

The experiment began when the broiler breeder hens were 49 weeks old. At the start of the experiment, each experimental enclosure contained 1386 hens and 130 roosters. The vertical distance between the perching platform and the slatted area was initially set at 35 cm. During weeks 50, 51, and 52, this distance was adjusted to 40, 45, and 50 cm, respectively. A Hikvision camera (1920 × 1080 p resolution, 25 FPS frame rate), connected to a video recorder, was positioned 1.0 m from the perching platform, with its field of view aligned to the platform’s height. Since the broiler breeder hens in this study had no prior experience using the perching platform at heights of 35, 40, 45, or 50 cm, an adaptation period was introduced to minimize the impact of early experiences on their spatial usage ability [27]. During the first four days of weeks 49, 50, 51, and 52, hens were allowed to adapt to the heights of perching platforms (35, 40, 45, and 50 cm). This adaptation period aimed to minimize any initial hesitation or discomfort that might have occurred due to the hens’ unfamiliarity with the different platform heights. By allowing the hens to adjust to the perching platforms before the actual video recording period, we ensured that their behavior during the recording days more accurately reflected their true jumping characteristics at the given heights, without being influenced by any prior inexperience with the platforms. Video recordings were conducted on days 5, 6, and 7 from 4:30 to 20:30.

### 2.3. Video Clip Screening and Data Acquisition Process

The following table outlines the detailed steps involved in the video analysis process used to study the jumping behavior of hens (Table 1).

**Figure 2:**
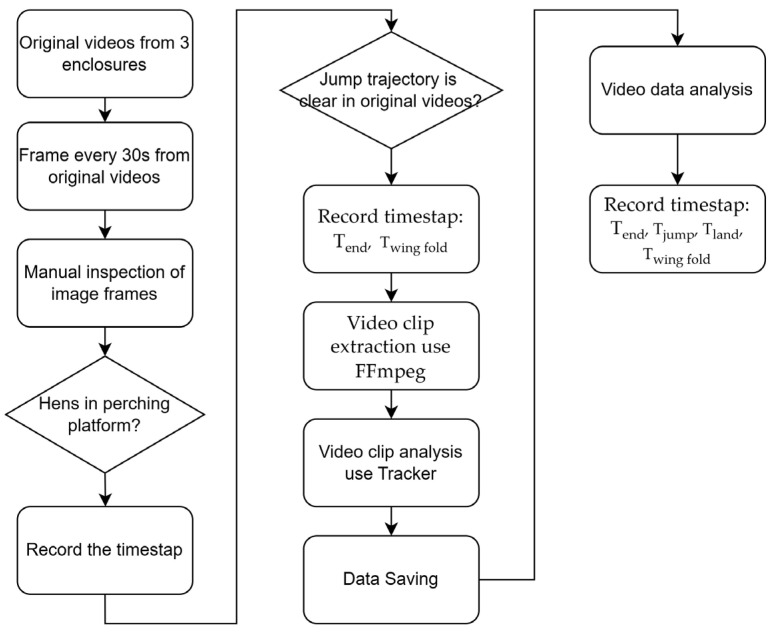
Video clip screening and data acquisition process.

The process followed is summarized as shown in the schematic diagram (Figure 2).

### 2.4. Jump Timing, Displacement, Angles, and Wing-Beating Frequencies

#### 2.4.1. Jump Timing

Based on these timestamps recorded in Section 2.4, the following metrics were calculated:

Take-off latency (*TL*): the time between $T_{end}$ and $T_{jump}$, which reflects the preparation time required for the hen to attempt a jump [28]. Jump duration (*JD*): the time between $T_{jump}$ and $T_{land}$, representing the duration of the entire jump [28]. Latency to achieve balance (*LAB*): The time between $T_{land}$ and $T_{wing\;fold}$, indicating the time required for the hen to achieve post-landing balance [21,28]. The specific calculation methods for each parameter are detailed below.

(1) $$TL=T_{jump}-T_{end}$$

(2) $$JD=T_{land}-T_{jump}$$

(3) $$LAB=T_{wing\;fold}-T_{land}$$

#### 2.4.2. Jump Displacement

Based on these timestamps and corresponding position coordinates of the hen’s head and body center at the given moment (t) recorded in Section 2.4, the following metrics were calculated:

Displacement during jumping-to-landing (*JL*) phase: the horizontal and vertical displacement of the body center and head during the JL phase, calculated using the coordinates of the body center and head marked in the image frames at $T_{jump}$ and $T_{land}$. Displacement during the jumping-to-balance (*JB*) phase: the horizontal and vertical displacement of the body center and head during the JB phase, determined from the coordinates marked in the image frames at $T_{jump}$ and $T_{wing\;fold}$. The specific calculation methods for each parameter are detailed below.

(4) $$∆X_{JL,k}=\left|X_{land}^{k}-X_{jump}^{k}\right|$$

(5) $$∆Y_{JL,k}=\left|Y_{land}^{k}-Y_{jump}^{k}\right|$$

(6) $$∆X_{JB,k}=\left|X_{b}^{k}-X_{jump}^{k}\right|$$

(7) $$∆Y_{JB,k}=\left|Y_{b}^{k}-Y_{jump}^{k}\right|$$

where $∆X_{JL,k}$ and $∆Y_{JL,k}$ represent the horizontal displacement and vertical displacement, respectively, of *k* (the head or body center of the broiler breeder hen) during the *JL* phase, cm. $∆X_{JB,k}$ and $∆Y_{JB,k}$ represent the horizontal displacement and vertical displacement, respectively, of *k* during the *JB* phase, cm. $X_{land}^{k}$ and $Y_{land}^{k}$ represent the horizontal and vertical coordinates of *k* in the image frame at $T_{land}$, while $X_{jump}^{k}$ and $Y_{jump}^{k}\;$ represent the horizontal and vertical coordinates of *k* in the image frame at $T_{jump}$. $X_{b}^{k}$ and $Y_{b}^{k}$ represent the horizontal and vertical coordinates of *k* in the image frame at $T_{wing\;fold}$.

#### 2.4.3. Jump Angles

Based on the horizontal and vertical displacement of broiler breeder hens during the *JL* phase, the jump angle (θ) was calculated as follows:

(8) $$\theta =Atan\frac{∆Y_{JD,k}}{∆X_{JD,k}}$$

#### 2.4.4. Wing-Beating Frequencies

Wing-beating is one of the muscular responses of birds to body balance disturbances [29]. In this study, the wing-beating frequencies were recorded for three phases: before jumping (WBB), during the jump duration (WBJD), and after landing (WBL).

### 2.5. Data Analysis and Processing

The data were organized using Microsoft Excel, and the Kolmogorov–Smirnov test was conducted to assess the normality of each indicator. The homogeneity of variances was evaluated using Levene’s test. *TL*, *JD*, and *LAB* data followed a normal distribution, with *JD* data exhibiting homogeneous variances. Multiple comparisons for *JD* were performed using ANOVA. Conversely, *TL* and *LAB* data showed non-homogeneous variances and were analyzed using the Kruskal–Wallis non-parametric test for independent samples. Jump displacement data and jump angle data followed a normal distribution with homogeneous variances, and ANOVA with LSD multiple comparisons was used for analysis. In contrast, the wing-beating frequencies did not follow normal distribution and were analyzed using the Kruskal–Wallis non-parametric test for independent samples. All results are expressed as mean ± standard deviation (SD, based on a sample size of 20), with significant difference at *p* < 0.05. Statistical analyses were performed using SPSS 25.0, and GraphPad Prism (version 8.0) was used to generate figures.

## 3. Results

### 3.1. Jump Timing and Wing-Beating Frequencies

**Figure 3:**
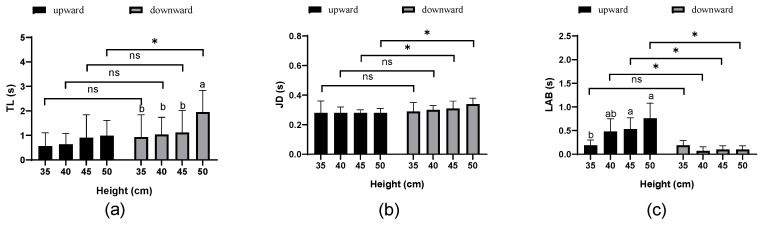
Jump timing characteristics at different perching platform heights and jump directions: (**a**) Take-off latency (*TL*); (**b**) jump duration (*JD*); (**c**) latency to achieve balance (*LAB*). Data represented as mean ± SD. The letters above the bars indicate differences between perching platform heights: identical letters represent no significant differences (*p* > 0.05), while different letters indicate significant differences (*p* < 0.05). An asterisk (*) denotes substantial differences between upward and downward jumps for the corresponding parameter (*p* < 0.05), whereas “ns” indicates no significant differences (*p* > 0.05).

Height had no significant effect on the take-off latency (*TL*) and jump duration (*JD*) for upward jumps (*p* = 0.253, 0.980, respectively), but it significantly influenced the latency to achieve balance (*LAB*) (*p* < 0.001). In contrast, height did not affect the *JD* or *LAB* for downward jumps (*p* = 0.065, 0.131, respectively), but it significantly affected *TL* (*p* = 0.016). Figure 3 illustrates the jump timing characteristics of broiler breeder hens at different perching platform heights and in upward and downward jump directions. For upward jumps from perching platforms at heights of 35, 40, 45, and 50 cm, *TL*, *JD*, and *LAB* exhibited an upward trend with increasing perching platform height; these changes were not statistically significant for *TL* and *JD* (*p* > 0.05). However, for upward jumps from 45 cm and 50 cm perching platforms, *LAB* was significantly higher than that for the 35 cm perching platform (*p* < 0.05). For downward jumps, *TL* at 50 cm was significantly higher than that at 35, 40, and 45 cm (*p* < 0.05).

The jump direction significantly affected *TL*, *JD*, and *LAB* (*p* < 0.05). At a height of 50 cm, *TL* for downward jumps was significantly higher than that for upward jumps (*p* = 0.004). The jump direction did not substantially affect *JD* at 35 cm and 40 cm (*p* = 0.908, 0.157, respectively). Still, it had a significant effect at 45 cm and 50 cm (*p* = 0.020, 0.005, respectively), with *JD* for downward jumps significantly exceeding that for upward jumps. At heights of 40, 45, and 50 cm, *LAB* for upward jumps was considerably higher than that for downward jumps (*p* = 0.000, 0.020, 0.005, respectively). On average, *TL* increased by 66.1%, *JD* by 10.7%, and *LAB* decreased by 76.5% for downward jumps compared to upward jumps at the same perching platform height (based on mean values) (Figure 3).

The perching platform height had no significant effect on the wing-beating frequencies before jumping (WBB) and after landing (WBL) (*p* = 0.135, *p* = 0.291, respectively). Still, it significantly influenced the wing-beating frequencies during the jump duration (WBJD) (*p* = 0.002). The jump direction significantly affected WBB and WBL (*p* < 0.05), while it had no significant effect on WBJD (*p* = 0.766). The interaction between the perching platform height and jump direction did not significantly affect WBB (*p* = 0.110) but significantly influenced WBJD and WBL (*p* = 0.024, *p* = 0.039, respectively). Table 2 shows that WBB was significantly higher for downward jumps than that for upward jumps from both 35 cm (*p* < 0.05) and 50 cm (*p* < 0.05) perching platforms. For upward jumps, WBJD and WBL increased as the perching platform height increased. At 50 cm, the WBL for upward jumps was significantly higher than that at 35 cm (*p* = 0.024). During downward jumps from the 50 cm, WBJD was considerably higher than that for upward jumps at the same height (*p* < 0.001). However, no statistically significant differences were observed for WBJD between upward and downward jumps at the perching platform heights of 35 cm, 40 cm, and 45 cm (*p* = 0.949, 0.413, and 0.347, respectively). Conversely, at heights of 40, 45, and 50 cm, WBL for downward jumps was significantly lower than that for upward jumps (*p* < 0.001).

### 3.2. Horizontal Displacement

Supplementary Table S2 presents the effects of various perching platform heights and jump directions on the horizontal displacement of body center (body) and head during *JL* and *JB* phase. The perching platform height significantly influenced the horizontal displacement of the body and head during the *JL* phase (*p* = 0.002, 0.001, respectively). The jump direction significantly affected the horizontal displacement of the body during both the *JL* and *JB* phases (*p* < 0.001). At the same time, their interaction had a significant effect on the body displacement during the *JL* phase (*p* = 0.023).

**Figure 4:**
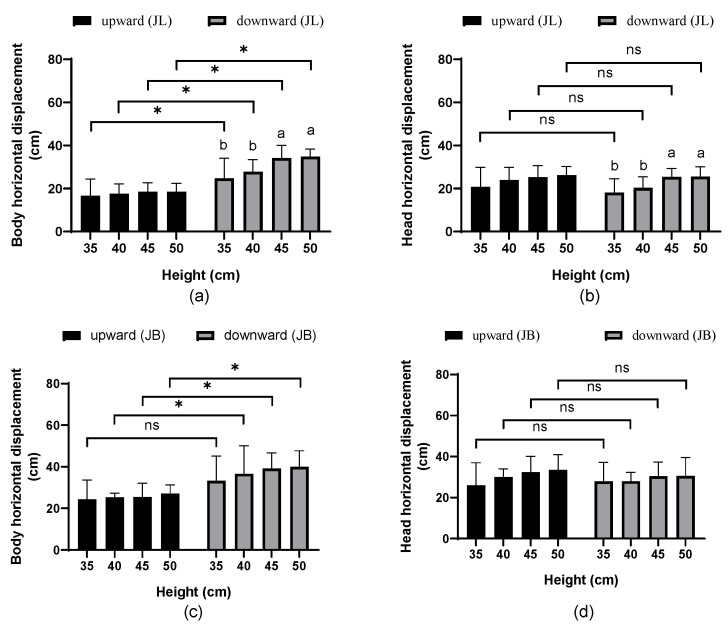
Horizontal displacement at different perching platform heights and jump directions: (**a**) Body horizontal displacement during the *JL* phase; (**b**) head horizontal displacement during the *JL* phase; (**c**) body horizontal displacement during the *JB* phase; (**d**) head horizontal displacement during the *JB* phase. Data represented as mean ± SD. The letters above the bars indicate differences between perching platform heights: identical letters represent no significant differences (*p* > 0.05), while different letters indicate significant differences (*p* < 0.05). An asterisk (*) denotes substantial differences between upward and downward jumps for the corresponding parameter (*p* < 0.05), whereas “ns” indicates no significant differences (*p* > 0.05).

Figure 4 shows the horizontal displacement of the body and head at different perching platform heights and jump directions. During the *JL* phase, the body and head displacements increased with the perching platform height. For upward jumps from heights of 35, 40, 45, and 50 cm, the body horizontal displacements were 16.7 ± 7.7, 17.6 ± 4.5, 18.5 ± 4.2, and 18.5 ± 3.9 cm, respectively (*p* > 0.05), while the head horizontal displacements were 20.7 ± 9.2, 23.9 ± 6.0, 25.2 ± 5.5, and 26.2 ± 4.1 cm, respectively (*p* > 0.05). For downward jumps at the same heights, body horizontal displacements were 24.8 ± 9.3, 27.8 ± 5.6, 34.1 ± 6.0, and 34.7 ± 3.6 cm, respectively, and head horizontal displacements were 18.1 ± 6.4, 20.3 ± 5.2, 25.4 ± 3.4, and 25.8 ± 4.6 cm, respectively. Compared to upward jumps during the *JL* phase, the body horizontal displacement of the downward jumps increased by an average of 70.3% (based on mean values). In comparison, the head horizontal displacement decreased by 6.7% (based on mean values), but this difference was not statistically significant (*p* > 0.05). At 45 cm and 50 cm heights, the body and head horizontal displacements during the downward jumps were significantly higher than those at 35 cm and 40 cm (*p* < 0.05). At the same perching platform height, the body horizontal displacement during the downward jumps was significantly greater than that during upward jumps (*p* < 0.05).

During the *JB* phase, both the body and head horizontal displacements increased as the perching platform height increased. For upward jumps from heights of 35, 40, 45, and 50 cm, the body horizontal displacements were 24.3 ± 9.4, 25.3 ± 2.0, 25.4 ± 6.7, and 27.1 ± 4.2 cm, respectively, while the head horizontal displacements were 26.1 ± 10.9, 30.0 ± 4.1, 32.4 ± 7.7, and 33.4 ± 7.5 cm, respectively (*p* > 0.05). For downward jumps, the body horizontal displacements were 33.3 ± 11.9, 36.6 ± 13.4, 39.2 ± 7.5, and 40.0 ± 7.7 cm, respectively, while the head horizontal displacements were 27.9 ± 9.3, 28.0 ± 4.3, 30.4 ± 6.9, and 30.5 ± 9.1 cm, respectively (*p* > 0.05). Compared to the upward jumps during the *JB* phase, the body horizontal displacement of the downward jumps increased by an average of 46.0%, and the head horizontal displacement increased by an average of 18.4% (based on mean values). At perching platform heights of 40, 45, and 50 cm, the body horizontal displacement during downward jumps to balance was significantly higher than that during upward jumps (*p* < 0.05) (Figure 4).

### 3.3. Jump Angles

Supplementary Table S2 presents the effects of various perching platform heights and jump directions on the body and head angles. The perching platform height had no significant effect on the body and head angles during the *JL* phase (*p* = 0.536, 0.281, respectively), while the jump direction significantly influenced both angles (*p* < 0.05). The interaction between the perching platform height and jump direction also significantly affected the body and head angles during the *JL* phase (*p* = 0.005, 0.011, respectively).

Table 3 shows the angles of the body and head at different perching platform heights and jump directions. Both the body and head angles increased as the perching platform height increased during upward jumps. In contrast, during downward jumps, both the body and head angles decreased as the perching platform height increased. At a perching platform height of 35 cm, the body angle during upward jumps was significantly lower than that at heights of 45 cm and 50 cm (*p* < 0.05). Additionally, at perching platform heights of 35 cm and 40 cm, the body angles during downward jumps were significantly higher than those at 50 cm (*p* < 0.05). The head angle during downward jumps at 35 cm was significantly higher than that at heights of 40, 45, and 50 cm (*p* < 0.05). At the same perching platform height, the body and head angles during downward jumps were, on average, 20.5% and 6.7% lower, respectively, compared to those during upward jumps (based on mean values). When the perching platform heights increased to 40, 45, and 50 cm, the body angles during downward jumps were significantly lower than those during upward jumps (*p* < 0.05). Similarly, at perching platform heights of 45 cm and 50 cm, the head angles during downward jumps were significantly lower than those during upward jumps (*p* < 0.05).

## 4. Discussion

### 4.1. Jump Timing and Wing-Beating Frequencies

Existing research indicates that in laying hen production systems, jumping behavior is influenced by the distance [24,30,31] and angles between perches [22,32,33], as well as the jump direction [22,28,34]. This study found that *TL* increased with the height of the perching platform, consistent with findings by Rufener et al., who observed similar effects of perch distance on *TL* in laying hens [26]. Compared to upward jumps, the significantly higher *TL* observed during downward jumps from the 50 cm perching platform suggests that greater perching platform heights require hens to take more time to adjust their posture and prepare for the jump. Similar phenomena have been reported in multiple studies [22,26,28,34,35], attributing the challenge of downward jumps to the increased difficulty of navigating to a lower perch [22,31]. For example, research has shown that the median time required to complete a downward jump increases significantly [22], and when the horizontal distance between perches is 60 cm with angles of 10° and 18°, *TL* for downward jumps is considerably higher than that for upward jumps [28]. Furthermore, as horizontal distances increase to 80 cm [28,34] and 1.15 m [34], laying hens are likelier to refuse downward jumps, exhibit clumsy landings, or miss the target perch entirely. In commercial flocks, laying hens transitioning downward to the litter area in single or multi-tier systems also show hesitation behaviors [35]. To mitigate these challenges, some researchers have introduced ramps, which over 79% of hens utilize [36]. However, studies also report that hesitation behaviors are more frequent during downward transitions using ramps than upward transitions [37].

Although the perching platform height did not significantly affect *JD* or *LAB* during downward jumps, it significantly affected *LAB* during upward jumps. For upward jumps from the perching platform at 45 cm and 50 cm, *LAB* was considerably higher than 35 cm, indicating that hens require more time to achieve body balance when landing on a higher perching platform. Additionally, WBJD and WBL for upward jumps increased with the height of the perching platform, suggesting that hens needed to beat their wings more frequently to complete upward jumps, land on higher perching platforms, and coordinate their bodies after landing [28]. This study also observed that at 35 cm and 50 cm perching platforms, the WBB of downward jumps was significantly higher than those of upward jumps. This could be attributed to hesitation behaviors before downward jumps, which aligns with findings by Moinard et al. [28].

### 4.2. Horizontal Distance

Current research on laying hens indicates that the frequency of jumps between perches decreases as the horizontal distance between them increases [30]. Laying hens have difficulty moving between perches more than 1.0 m apart, as their jumping ability is limited by a threshold distance [30]. When the distance between perches exceeds 50 cm, it impairs the hens’ navigation ability, negatively affecting their welfare and health [22,24,28]. Accordingly, some studies recommend that the distance between perches in laying hen production systems should not exceed 50 cm [26]. In this study, horizontal distances were not designed; instead, different heights of the perching platform were provided, allowing hens to select the points of take-off and landing freely. During the *JL* phase, at the perching platform heights of 35, 40, 45, and 50 cm, the body and head horizontal displacements during upward jumps ranged from 16.7 ± 7.7 to 18.5 ± 4.2 cm and from 20.7 ± 9.2 to 26.2 ± 4.1 cm, respectively. The body and head horizontal displacements of the downward jump ranged from 24.8 ± 9.3 to 34.7 ± 3.6 cm and from 18.1 ± 6.4 to 25.8 ± 4.6 cm, respectively. Notably, both the body and head horizontal displacements during both upward and downward jumps did not exceed 50 cm, aligning with the recommendations for the horizontal distance between perches for laying hens.

In this study, both the body and head horizontal displacement during the *JL* and *JB* phases increased as the height of the perching platform increased, indicating a correlation between the perching platform height and horizontal displacement when hens freely selected their take-off and landing points. During the *JL* phase of downward jumps, the horizontal displacement of the body and head at 45 and 50 cm was significantly higher than that at 35 and 40 cm. Additionally, the body horizontal displacement during downward jumps in the *JL* phase was considerably greater than that during upward jumps at all perching platform heights. Hens required a certain distance to maintain body balance after landing. During the *JB* phase, at perching platform heights of 40, 45, and 50 cm, the body horizontal displacement during downward jumps was significantly greater than that during upward jumps. These results suggest that as the vertical distance between the take-off and landing points increases, hens require a greater horizontal distance to achieve balance, with downward jumps requiring a longer distance than upward jumps.

Interestingly, the jump direction did not significantly affect the head horizontal displacement during the *JL* and *JB* phases. This could be because hens extend their heads toward the landing point to explore before jumping, particularly during downward jumps, when the head is extended further toward the landing point [38]. After landing, the head position is inconsistent with the pre-jump state, which may introduce variability in the head horizontal displacement compared to the body horizontal displacement. To ensure successful landing and maintain body balance after landing, when the vertical distance between perching platforms is 35–50 cm, the horizontal distance between them should be between 33.3 ± 11.9 cm and 40.0 ± 7.7 cm.

### 4.3. Jump Angles

In laying hens, an increase in the angle between perches significantly raises the difficulty of downward jumps [22] and prolongs the take-off latency (*TL*) [26,31]. To minimize the risk of injury, studies have recommended that the angle between perches of different heights should not exceed 45°, and both horizontal and vertical distances should be minimized to facilitate downward movement [22]. Other studies have found that when the angle between perches reaches 45° or 60°, the take-off latency (*TL*) for downward jumps in laying hens can be extended by up to 10 min [32]. Scholz et al. (2014) suggested that the optimal vertical distance for downward jumps in laying hens is 50 cm with a slope of 34°, beyond which the proportion of safe landings decreases significantly [33]. Additionally, other studies have proposed that the angle between perches in laying hen systems should not exceed 30° [26]. Previous research has shown that as the angle between perches increases, the number of hens unable to complete downward jumps also rises [22]. Therefore, designing appropriate angles between perching platforms in broiler breeder systems is crucial, particularly for ensuring safe downward jumps in hens. Although our study did not examine varying gradients of jumping angles for breeders, the horizontal and vertical displacement during the *JL* phase collectively determined the broiler breeder hens’ jumping angles (head and body) when allowed to jump freely. In our study, during upward jumps to land, both the body and head angles increased as the height of the perching platform rose, ranging from 52.4° ± 14.5° to 63.5° ± 6.1° for body angles and from 36.8° ± 7.0° to 39.2° ± 4.4° for head angles. Previous research has indicated that poultry can successfully jump upward when the angle between perches is 60°, but *TL* is significantly higher compared to that at angles of 0°, 30°, or 45° [22]. In this study, the broiler breeder hens exhibited a jumping angle of 60.2° ± 7.7° at a perching platform height of 45 cm, which exceeds the recommended values from previous studies [22]. However, no significant increase in *TL* was observed until the perching platform height reached 50 cm, with a jumping angle of 63.5° ± 6.1°. This could be attributed to the flexibility in this study, where hens could freely select their take-off and landing points, enhancing their ability to control their body movements.

During downward jumps, both the body and head angles decreased as the height of the perching platform increased, consistent with findings from other studies [38]. For downward jumps at perching platform heights of 35, 40, 45, and 50 cm, the body angles ranged from 42.2° ± 5.4° to 49.5° ± 7.0°, while the head angles ranged from 33.4° ± 5.6° to 40.9° ± 6.4°. Moreover, both the body and head angles during downward jumps to landing were lower than those during upward jumps. These results suggest that broiler breeder hens adopt more conservative angles during downward jumps to reduce the landing impact than those adopted during upward jumps. In this study, the body angles during downward jumps exceeded the recommended 45° threshold proposed by Scott et al. [22] and Lambe et al. [32], while the head angles aligned with the 34° safety angle for downward jumps in laying hens reported by Scholz et al. (2014) [33].

Considering that the changes in head angles during the jumping process of broiler breeder hens in this study may be influenced by their exploratory behavior on the perching platform [38], we recommend designing perching facilities based on the body center angles during the hens’ jumps. In addition to the impact of the perching platform height on these angles, the jump direction significantly affects the angles at platform heights of 40, 45, and 50 cm. Therefore, taking into account the behavioral needs of broiler breeder hens during upward and downward jumps between perches, and integrating existing research on the jumping angles of laying hens, we suggest that the angle for upward jumps should be kept below 60°, while the angle for downward jumps should range from 42.2° to 49.5°. Considering both upward and downward jumping angles, the angle between perches should be maintained within the range of 42.2° to 49.5°. This recommended range is similar to the angle of 45° suggested by Scott et al. [22] in their study on jumping angles in laying hens.

### 4.4. Recommendations for Perch Facility Design

The findings indicate that in broiler breeder systems, to ensure safe jumping and landing, the vertical distance between perching platforms should be limited to below 50 cm, and the horizontal distance between platforms should be kept between 33.3 cm and 40.0 cm when no auxiliary navigation facilities are present. This recommended range is smaller than the horizontal distances reported for laying hens in existing studies [28]. For higher perching platforms (≥45 cm), optimizing the angles between platforms is recommended to reduce awkward landings and minimize balance loss during jumps. The angle between perches should be maintained within the range of 42.2° to 49.5°, which is similar to the angle of 45° suggested by Scott et al. [22]. Additionally, providing ramps between platforms may effectively reduce the take-off latency and mitigate risks associated with downward jumps [35,39].

### 4.5. Limitations and Future Directions

Before the initiation of this study (prior to 49 weeks of age), another study identified aggressive mating behaviors within the flock. Recognizing that the introduction of perching platforms could potentially alleviate welfare issues related to mating, this context led us to introduce perching platforms with heights exceeding 30 cm only at 49 weeks of age (with a 30 cm high platform area already present in the enclosures). Considering that early exposure to perching facilities may influence the hens’ perching behavior, future research could attempt to introduce perches at an earlier developmental stage to further investigate their long-term impact on the behavioral development of broiler breeders.

This study also did not involve individually marking the hens. Future studies should consider implementing an individual marking system to optimize data collection methods and enhance the operability of individual behavior tracking.

This study mainly focused on mechanical and motion parameters, without directly measuring welfare outcomes (e.g., injury rates or stress indicators). Future research could integrate perching facility design parameters with welfare indicators to further optimize perch designs and improve the overall welfare of broiler breeders, promoting the sustainability of poultry production systems.

## 5. Conclusions

This study explored the jumping characteristics of broiler breeder hens during voluntary jumps from perching platforms of varying heights (35, 40, 45, and 50 cm). The key findings indicate that both the take-off latency (*TL*) and jump duration (*JD*) for both upward and downward jumps increased as the height of the perching platform rose, with downward jumps showing a longer *TL* and *JD* compared to those during upward jumps. Additionally, hens exhibited more conservative angles during downward jumps. The results highlight the significant influence of the platform height and jump direction on jump timing, displacement, and angles. Notably, the body horizontal displacement of downward jumps was significantly higher than that during upward jumps during the *JL* period, and the body and head angles decreased as the height increased during downward jumps. These findings underscore the importance of considering both the height and the design features of perching facilities, such as angles and horizontal displacements, to ensure safe and efficient jumping behavior. To improve the welfare of broiler breeders, perching platform designs should focus on optimizing these parameters, ensuring that both upward and downward jumps can be performed safely and comfortably. By applying these insights, perching systems can be better designed to enhance the overall welfare of broiler breeder hens, particularly in minimizing risks associated with jumping behaviors.

## Figures and Tables

**Table 1:** Sequential Steps in Video Analysis for Hen Jump Observations.

Step	Description
1. Preliminary Video Screening	(1) One frame was extracted every 30 s from the recorded videos in three experimental enclosures. (2) Each frame was manually checked by one observer to confirm whether any hens were utilizing the perching platform. (3) When hens were observed utilizing the perching platform, the corresponding timestamp of the behavior was recorded by one observer.
2. Selection of Valid Video Segment	(1) Based on the recorded timestamps, the original video was revisited by one observer to locate and confirm the relevant segments where hens used the perching platform. The following criteria were applied to filter the video clips: a. Ensure the target hen is not obstructed by other chickens surrounding the perching platform. b. Ensure that the target hen’s jumping trajectory is not obstructed by the edge of the perching platform during take-off or landing. To ensure clear tracking of the jumping trajectory, only video clips where the trajectory is clearly visible and occurs within the camera’s fully observable region were selected. The trajectory must be entirely within the camera’s field of view, meaning that the hen jumps or lands from the edge of the perching platform near the slatted area, with the edge being perpendicular to the camera’s viewing plane. (2) Timestamp recording: if the video meets the selection criteria, the starting timestamp for the period from the hens facing the landing platform to the completion of the jump and balance maintenance was recorded by one observer. (3) Limitation on video segments: For the 50 cm height perching platform, only 20 valid video clips were collected due to video quality and experimental constraints. This limitation was not only due to the obstructed view of the target hens but also because of their jumping characteristics. Specifically, when hens jumped from the edge of the perching platform parallel to the camera, the camera could not capture the complete jumping trajectory. Moreover, some hens landed in the litter area, making it difficult for the camera to capture the required behavioral data. Therefore, to ensure consistency of data across different platform heights, 20 video clips of both upward and downward jumps were selected at each of the following heights: 35 cm, 40 cm, 45 cm, and 50 cm.
3. Video Clip Extraction	Based on the recorded timestamps, FFmpeg was used to extract the corresponding video segments from the original video, covering the entire jump process from the hens facing the landing platform to the completion of the jump and balance maintenance.
4. Video Clip Analysis	(1) The extracted video clips were imported into Tracker software (version 6.2.0). (2) The step size for video analysis was set to 1 frame to ensure frame-by-frame tracking of the hen’s movement. (3) Calibration mark: a calibration mark was set using parallel lines on the vertical rods of the perching platform at 5 cm intervals as a reference. (4) Length calibration: the calibration tool was used to standardize the length by calibrating the calibration mark against the actual physical dimensions of the perching platform (35 cm, 40 cm, 45 cm, and 50 cm), ensuring a relative error of ±0.9%. (5) Setting the coordinate origin and axis angle: The coordinate origin was set at the intersection of the vertical rod near the camera and the slatted area. The *Y*-axis was aligned parallel to the vertical rod. (6) Marking the target broiler breeder: A new point was created and named “head”, and the eye position of the target hens was manually marked frame-by-frame by one observer. A new RGB region was created with a rectangular shape. The width and length of the rectangle were adjusted to fit the body of the hens (excluding the head, neck, and legs). The center of the rectangle represented the center of the hen’s body. The center of the body was manually marked frame-by-frame to ensure accurate reflection of the hen’s position in each frame. And then, Tracker software recorded the timestamp of each frame (e.g., frame 1 at 0 s, frame 2 at 0.04 s…) and the corresponding x and y coordinates. (7) Data saving: once the marking was complete, the data file was manually exported and saved.
5. Video Data Analysis	In Tracker software, the video file was cross-checked by one observer with the exported data to record the following parameters: the end of the hen’s final foot adjustment before facing the land point $\left(T_{end}\right)$, the time when the jump started $\left(T_{jump}\right)$, the time when both feet touched the land point $\left(T_{land}\right)$, the time when the wings were fully folded after landing $(T_{wing\;fold}$), and the corresponding position coordinates of the hen’s head and body center at the given moment (t).

**Table 2:** Wing-beating frequency characteristics.

Perching Platform Height (cm)	Categories	Upward Jump	Downward Jump	*p*-Value
35	WBB	0.00	0.60 ± 0.61	0.014
40		0.00	0.33 ± 0.47	0.164
45		0.17 ± 0.55	0.60 ± 0.49	0.069
50		0.00	0.43 ± 0.49	0.000
35	WBJD	1.62 ± 0.49	1.50 ± 0.82 ^b^	0.949
40		1.64 ± 0.64	1.47 ± 0.81 ^b^	0.413
45		1.83 ± 0.80	1.45 ± 0.50 ^b^	0.347
50		1.91 ± 0.51	2.75 ± 0.83 ^a^	0.000
35	WBL	0.77 ± 2.50 ^bc^	0.47 ± 0.62 ^a^	0.451
40		1.64 ± 1.61 ^ab^	0.07 ± 0.25 ^b^	0.008
45		1.17 ± 1.86 ^ab^	0.00 ^b^	0.016
50		2.82 ± 2.37 ^a^	0.00 ^b^	0.000

Note: WBB represents wing-beating frequencies before jumping, WBJD represents wing-beating frequencies during the jump duration, and WBL represents wing-beating frequencies after landing. Superscript letters indicate differences between perching platform heights for each parameter: identical letters denote no significant differences (*p* > 0.05), while different letters represent significant differences (*p* < 0.05). *p*-values compare differences between upward and downward jumps: *p* < 0.05 indicates an important difference, while *p* > 0.05 indicates no significant difference.

**Table 3:** Jump angles at different perching platform heights and jump directions.

Categories	Perching Platform Height (cm)	Upward Jump (°)	Downward Jump (°)	*p*-Value
Body	35	52.4 ± 14.5 ^b^	49.5 ± 7.0 ^a^	0.499
	40	59.6 ± 8.8 ^ab^	47.9 ± 4.6 ^a^	0.000
	45	60.2 ± 7.7 ^a^	47.8 ± 5.4 ^ab^	0.000
	50	63.5 ± 6.1 ^a^	42.2 ± 5.4 ^b^	0.000
head	35	36.8 ± 7.0	40.9 ± 6.4 ^a^	0.174
	40	38.2 ± 4.5	34.3 ± 5.0 ^bc^	0.074
	45	38.7 ± 3.8	34.1 ± 2.7 ^bc^	0.006
	50	39.2 ± 4.4	33.4 ± 5.6 ^c^	0.029

Note: Superscript letters and *p*-values indicate the same meanings as those in Table 2.

## Data Availability

The data will be made available on reasonable request of the corresponding author.

## Supplementary Materials

The following supporting information can be downloaded at: https://www.mdpi.com/article/10.3390/ani15050725/s1, Table S1. Nutritional Composition of Feed for AA+ broiler breeders. Table S2. Effects of perching platform height, jump direction.

## References

[1] van Emoes R.A., de Jong I.C. Promising Management Measures to Solve Welfare Problems in Broiler Breeders. Proceedings of the 2nd International Poultry Meat Congress.

[2] Millman S.T., Duncan I.J.H., Widowski T.M. (2000). Male Broiler Breeder Fowl Display High Levels of Aggression toward Females. Poult. Sci..

[3] De Jong I.C., Guémené D. (2011). Major Welfare Issues in Broiler Breeders. Worlds. Poult. Sci. J..

[4] Leone E.H., Estévez I. (2008). Economic and Welfare Benefits of Environmental Enrichment for Broiler Breeders. Poult. Sci..

[5] van den Oever A.C.M., Kemp B., Rodenburg T.B., Van de Ven L.J.F., Bolhuis J.E. (2021). Gregarious Nesting in Relation to Floor Eggs in Broiler Breeders. Animal.

[6] Gebhardt-Henrich S.G., Jordan A., Toscano M.J., Würbel H. (2020). The Effect of Perches and Aviary Tiers on the Mating Behaviour of Two Hybrids of Broiler Breeders. Appl. Anim. Behav. Sci..

[7] Olsson I.A.S., Keeling L.J. (2000). Night-Time Roosting in Laying Hens and the Effect of Thwarting Access to Perches. Appl. Anim. Behav. Sci..

[8] Schrader L., Müller B. (2009). Night-Time Roosting in the Domestic Fowl: The Height Matters. Appl. Anim. Behav. Sci..

[9] Appleby M.C. (1995). Perch Length in Cages for Medium Hybrid Laying Hens. Br. Poult. Sci..

[10] Vasdal G., Gebhardt-Henrich S.G., Tahamtani F., Kittelsen K.E. (2022). Effect of Perch Access on Perching, Health and Production Outcomes in Commercial Broiler Breeder Flocks. Poult. Sci..

[11] Gebhardt-Henrich S.G., Toscano M.J., Würbel H. (2017). Perch Use by Broiler Breeders and Its Implication on Health and Production. Poult. Sci..

[12] Gebhardt-Henrich S.G., Toscano M.J., Würbel H. (2018). Use of Aerial Perches and Perches on Aviary Tiers by Broiler Breeders. Appl. Anim. Behav. Sci..

[13] Riber A.B., de Jong I.C., van de Weerd H.A., Steenfeldt S. (2017). Environmental Enrichment for Broiler Breeders: An Undeveloped Field. Front. Vet. Sci..

[14] Zuidhof M.J., Schneider B.L., Carney V.L., Korver D.R., Robinson F.E. (2014). Growth, Efficiency, and Yield of Commercial Broilers from 1957, 1978, and 2005. Poult. Ence.

[15] Vasdal G., Gebhardt-Henrich S.G., Tahamtani F., Kittelsen K.E. (2022). Perch Use in Commercial Broiler Breeders—Preference for Perch Material and Effect of Age. Appl. Anim. Behav. Sci..

[16] Giersberg M.F., Kemper N., Spindler B. (2020). Provision of Perches and Their Use by Broiler Breeders on the Basis of a Case Study. Eur. Poult. Sci..

[17] Li B., Wang Y., Rong L., Zheng W. (2023). Research Progress on Animal Environment and Welfare. Anim. Res. One Health.

[18] Rufener C., Makagon Maja M. (2020). Keel Bone Fractures in Laying Hens: A Systematic Review of Prevalence across Age, Housing Systems, and Strains. J. Anim. Sci..

[19] Heerkens J.L.T., Delezie E., Rodenburg T.B., Kempen I., Zoons J., Ampe B., Tuyttens F.A.M. (2016). Risk Factors Associated with Keel Bone and Foot Pad Disorders in Laying Hens Housed in Aviary Systems. Poult. Sci..

[20] Heerkens J.L.T., Delezie E., Ampe B., Rodenburg T.B., Tuyttens F.A.M. (2016). Ramps and Hybrid Effects on Keel Bone and Foot Pad Disorders in Modified Aviaries for Laying Hens. Poult. Sci..

[21] Moinard C., Rutherford KM D., Haskell M.J. (2005). Effects of Obstructed Take-Off and Landing Perches on the Flight Accuracy of Laying Hens. Appl. Anim. Behav. Sci..

[22] Scott G.B., Lambe N.R., Hitchcock D. (1997). Ability of Laying Hens to Negotiate Horizontal Perches at Different Heights, Separated by Different Angles. Br. Poult. Sci..

[23] Scott G.B., Parker C.A.L. (1994). The Ability of Laying Hens to Negotiate between Horizontal Perches. Appl. Anim. Behav. Sci..

[24] Taylor P.E., Scott G.B. (2003). The Ability of Laying Hens to Negotiate Jumps between Horizontal Perches at Different Light Intensities. Appl. Anim. Behav. Sci..

[25] Taylor P.E., Scott G.B., Rose S.P. (2003). Ability of Laying Hens to Negotiate Jumps between Horizontal Perches: Effects of Light Intensity and Perch Colour. Br. Poult. Sci..

[26] Rufener C., Rentsch A.K., Stratmann A., Toscano M.J. (2020). Perch Positioning Affects Both Laying Hen Locomotion and Forces Experienced at the Keel. Animals.

[27] Pullin A.N., Rufener C.B., Millman S.T., Tarlton J.F., Toscano M.J., Blatchford R.A., Makagon M.M. (2024). Providing Elevated Structures in the Pullet Rearing Environment Affects Behavior during Initial Acclimation to a Layer Aviary. Poult. Sci..

[28] Moinard C., Statham P., Haskell M.J., Mccorquodale C., Jones R.B., Green P.R. (2004). Accuracy of Laying Hens in Jumping Upwards and Downwards between Perches in Different Light Environments. Appl. Anim. Behav. Sci..

[29] Necker R., Janßen A., Beissenhirtz T. (2000). Behavioral Evidence of the Role of Lumbosacral Anatomical Specializations in Pigeons in Maintaining Balance during Terrestrial Locomotion. J. Comp. Physiol. A.

[30] Scott G.B., Hughes B.O., Lambe N.R. (1999). Ability of Laying Hens to Jump between Perches: Individual Variation and the Effects of Perch Separation and Motivation on Behaviour. Br. Poult. Sci..

[31] Taylor P.E., Scott G.B., Paul R. (2003). The Ability of Domestic Hens to Jump between Horizontal Perches: Effects of Light Intensity and Perch Colour. Appl. Anim. Behav. Sci..

[32] Lambe N.R., Scott G.B., Hitchcock D. (1997). Behaviour of Laying Hens Negotiating Perches at Different Heights. Anim. Welf..

[33] Scholz B., Kjaer J.B., Schrader L. (2014). Analysis of Landing Behaviour of Three Layer Lines on Different Perch Designs. Br. Poult. Sci..

[34] Moinard C., Statham P. (2004). Control of Landing Flight by Laying Hens: Implications for the Design of Extensive Housing Systems. Br. Poult. Sci..

[35] Pettersson I.C., Weeks C.A., Nicol C.J. (2017). The Effect of Ramp Provision on the Accessibility of the Litter in Single and Multi-Tier Laying Hen Housing. Appl. Anim. Behav. Sci..

[36] Toscano M.J., Jalali A.S., Siegford J.M., Stratmann A. (2024). Providing Ramps during Lay Has Larger Impacts on Laying Hens than Ramps at Rearing. Poult. Sci..

[37] Pettersson I.C., Weeks C.A., Norman K.I., Nicol C.J. (2017). The Ability of Laying Pullets to Negotiate Two Ramp Designs as Measured by Bird Preference and Behaviour. PeerJ.

[38] Moinard C., Rutherford K.M.D., Statham P., Green P.R. (2005). Visual Fixation of a Landing Perch by Chickens. Exp. Brain Res..

[39] Stratmann A., Fröhlich E.K.F., Gebhardt-Henrich S.G., Harlander-Matauschek A., Würbel H., Toscano M.J. (2015). Modification of Aviary Design Reduces Incidence of Falls, Collisions and Keel Bone Damage in Laying Hens. Appl. Anim. Behav. Sci..

