# Supplementary material for: Jumping Characteristics of Broiler Breeder Hens at Different Perching Platform Heights
Source: Animals (Basel). 2025 Mar 3;15(5):725. doi: 10.3390/ani15050725 (PMC11898682; doi:10.3390/ani15050725)
Supplement: Supplementary file 1 [file animals-15-00725-s001.zip › animals-3488282-supplementary.pdf]

Table S1. Nutritional Composition of Feed for AA+ broiler breeders.

Nutrient	40 Weeks and Above (Laying Period)
Crude Protein	15-18%
Crude Fiber	3-5%
Calcium	3.5-4.0%
Phosphorus	0.5-0.7%
Amino Acids (Lysine)	0.7-0.9%
Energy (ME)	2800-3000 Kcal/kg
Sodium	0.15-0.20%
Chlorine	0.2-0.3%
Vitamin A	3000-4000 IU/kg
Vitamin D3	800-1000 IU/kg
Vitamin E	40-60 mg/kg
Selenium	0.15-0.2 mg/kg

Note: The nutritional composition values are based on the dietary requirements for AA+ broiler breeders at 40 weeks and above during the laying period. Adjustments may be necessary based on factors such as weight, health conditions, and environmental factors.

Table S2. Effects of perching platform height, jump direction

Factors	Dependent Variable	F	d.f.	P-value
Height of perching platform	horizontal displacement of the body center (body) during JL	5.260	3	0.002
	horizontal displacement of the head during JL	5.721	3	0.001
	horizontal displacement of the body center (body) during JB	1.575	3	0.202
	horizontal displacement of the head during JB	1.524	3	0.216
	Body angle	0.731	3	0.536
	Head angle	1.297	3	0.281
Jump direction	horizontal displacement of the body center (body) during JL	84.528	1	0.000
	horizontal displacement of the head during JL	1.711	1	0.194
	horizontal displacement of the body center (body) during JB	38.383	1	0.000
	horizontal displacement of the head during JB	0.278	1	0.600
	Body angle	46.680	1	0.000
	Head angle	4.916	1	0.029
(Height of perhing platform) * (Jump direction)	horizontal displacement of the body center (body) during JL	3.334	3	0.023
	horizontal displacement of the head during JL	0.811	3	0.491
	horizontal displacement of the body center (body) during JB	1.014	3	0.319
	horizontal displacement of the head during JB	0.190	3	0.913
	Body angle	4.580	3	0.005
	Head angle	3.938	3	0.011
